# In Situ Encapsulation of Camptothecin by Self-Assembly of Poly(acrylic acid)-*b*-Poly(*N*-Isopropylacrylamide) and Chitosan for Controlled Drug Delivery

**DOI:** 10.3390/polym15112463

**Published:** 2023-05-26

**Authors:** Yi-Cheng Huang, Yang-Jie Zeng, Yu-Wei Lin, Hung-Chih Tai, Trong-Ming Don

**Affiliations:** 1 Department of Food Science, National Taiwan Ocean University, No. 2, Beining Rd., Zhongzheng Dist., Keelung City 202301, Taiwan; ychuang@email.ntou.edu.tw (Y.-C.H.); jk7041191k@gmail.com (Y.-J.Z.); tai.udn.george@gmail.com (H.-C.T.); 2 Department of Chemical and Materials Engineering, Tamkang University, No. 151 Yingzhuan Rd., Tamsui Dist., New Taipei City 251301, Taiwan; odinsg2007@gmail.com

**Keywords:** stimuli-responsive polymers, poly(acrylic acid), poly(*N*-isopropylacrylamide), chitosan, camptothecin, drug delivery

## Abstract

Camptothecin (CPT) has been shown to exhibit anticancer activity against several cancers. Nevertheless, CPT is very hydrophobic with poor stability, and thus its medical application is limited. Therefore, various drug carriers have been exploited for effectively delivering CPT to the targeted cancer site. In this study, a dual pH/thermo-responsive block copolymer of poly(acrylic acid-*b*-*N*-isopropylacrylamide) (PAA-*b*-PNP) was synthesized and applied to encapsulate CPT. At temperatures above its cloud point, the block copolymer self-assembled to form nanoparticles (NPs) and in situ encapsulate CPT, owing to their hydrophobic interaction as evidenced by fluorescence spectrometry. Chitosan (CS) was further applied on the surface through the formation of a polyelectrolyte complex with PAA for improving biocompatibility. The average particle size and zeta potential of the developed PAA-*b*-PNP/CPT/CS NPs in a buffer solution were 168 nm and −30.6 mV, respectively. These NPs were still stable at least for 1 month. The PAA-*b*-PNP/CS NPs exhibited good biocompatibility toward NIH 3T3 cells. Moreover, they could protect the CPT at pH 2.0 with a very slow-release rate. At pH 6.0, these NPs could be internalized by Caco-2 cells, followed by intracellular release of the CPT. They became highly swollen at pH 7.4, and the released CPT was able to diffuse into the cells at higher intensity. Among several cancer cell lines, the highest cytotoxicity was observed for H460 cells. As a result, these environmentally-responsive NPs have the potential to be applied in oral administration.

## 1. Introduction

Camptothecin (CPT) is a hydrophobic drug obtained from the isolation of bark and stem of Camptotheca acuminata, which has been used in traditional Chinese medicine [1,2]. It has been shown to exhibit anticancer activity against breast, ovarian, colon, lung, and stomach cancers [3,4,5]. CPT has a planar pentacyclic ring structure, which is considered to be one of the most important factors in topoisomerase inhibition [6]. CPT can bind to the topoisomerase I and DNA complex to inhibit DNA replication and RNA transcription processes, thereby resulting in apoptosis [7]. However, there are several disadvantages for the CPT, including poor aqueous solubility and stability. Therefore, water-soluble CPT derivatives, such as irinotecan, topotecan, and 9-aminocamptothecin have been synthesized, which are currently approved for the treatment of ovarian, cell lung cancer, colorectal carcinoma refractory, and other metastatic colorectal cancers [8,9]. However, these CPT derivatives still suffer from several important drawbacks mainly related to the poor stability of the lactone ring, the short half-life of the compounds in blood, and a number of non-resolved toxic effects. Consequently, the development of controlled delivery strategies could lead to significant advantages in the clinical use of CPT. In this regard, various drug carriers have been exploited for effectively delivering CPT to the targeted cancer site, such as micelles, liposomes, polymer conjugates, and self-assembled molecules, etc. [10,11,12,13,14,15,16,17]. Zhang et al. [18] prepared CPT-loaded micelles based on disodium glycyrrhizin (Na2GA) and tannic acid (TA). The CPT-loaded micelles (equivalent to 5 mg/kg CPT) showed a significant inhibition of tumor growth, nearly 93.3% tumor inhibitory ratio. Lee et al. [19] prepared indocyanine green (ICG) and CPT co-loaded perfluorocarbon double-layer nanocomposite named ICPNC for breast cancer therapy. In particular, nanoparticles (NPs) can provide an excellent platform for overcoming these challenges, owing to their simultaneous delivery of drugs to targeted cells, synergistic therapeutic effects, and suppression of drug resistance.

Recently, environmentally-responsive polymers have been applied as carriers for controlled drug release [20,21,22,23,24]. Among them, thermo-responsive polymers which can undergo phase transition in solvent at a certain critical temperature have been extensively studied [25,26,27], apart from pH-responsive polymers [28,29]. One of the most studied thermo-responsive polymers is poly(*N*-isopropylacrylamide) (PNP), which has a lower critical solution temperature (LCST) at 32 °C in water [30,31,32,33]. Heskins and Guillet [30] pioneered in the study of solution properties of PNP and found that the observation of LCST was due to an entropy effect. Below LCST, relatively strong hydrogen bonds are formed between water and PNP in the solution, resulting in a more ordered structure. As the temperature is raised, breakage of hydrogen bonds occurs while the hydrophobic interaction among isopropyl groups and intermolecular hydrogen bonds increase; therefore, this leads to the precipitation and phase separation. This property makes it useful for drug delivery applications, where it can be used to control the release of drugs in response to changes in temperature. In addition, the transition temperature and other properties can be tuned by copolymerization of *N*-isopropylacrylamide (NIPAAm) with other monomers [34,35,36,37,38,39]. The structure of the formed micellar aggregates can also be manipulated by changing the environmental conditions. Zhao et al. [40] synthesized a block copolymer of poly(*N*-isopropylacrylamide)-*b*-poly(L-lysine) (PNP-*b*-PLL) by ring-opening polymerization. These PNP-*b*-PLL copolymers could self-assemble into micelle-like aggregates with PNP as the hydrophobic block in the environments of acidic pH and high temperatures, while at alkaline pH and low temperatures they self-assemble into particles with PLL as the hydrophobic block. Many researchers have developed temperature-responsive hydrogels containing PNP for various applications, such as tissue engineering, wound healing, and drug delivery [25,26,27]. It has shown promising results in various preclinical studies. However, further research is still needed to fully understand its potential for use in drug delivery and other medical applications. It has already been proven that PNP and some of its copolymers are biocompatible with many cell lines, exhibiting no significantly cytotoxic and genotoxic responses to human keratinocyte (HaCaT), colon cells (SW 480), murine preadipose cells (3T3-L1), human embryonic kidney cells (HEK293), human carcinoma-derived cells (A549), human cervical cancer cells (HeLa), human bronchial epithelial (HBE), macrophages RAW264.7, and human umbilical vein endothelial cells (HUVEC) [41,42,43,44,45]. For example, Kim et al. [41] prepared dual-targeted stimuli-responsive drug carrier comprised of PNP hydrogel containing magnetite nanoparticles (MNP) and further conjugated with folic acid as a targeting ligand for HeLa cancer cells. Their results showed that the PNP hydrogel showed little to no cytotoxicity. Nevertheless, after loading with anticancer drug DOX, they found a synergistically enhanced intracellular uptake by HeLa cells in vitro enabled by both magnetic attraction and receptor-mediated endocytosis. Vihola et al. [45] showed that thermo-responsive polymers, such as PNP and poly(*N*-vinylcaprolactam) (PVCL) were well-tolerated at concentrations below 10.0 mg/mL in Caco-2 and Calu-3 cell cultures. Nonetheless, a positive correlation between concentration and cytotoxicity was found in the monomers.

Acrylic acid (AA) is an easily available and inexpensive monomer. Its polymer form, poly(acrylic acid) (PAA), is a commonly used polymer in various medical applications, including wound dressings, drug delivery, and oral care products [46,47]. Intensive research of wound dressings and adhesive products that contain PAA as a component for use in the management of various types of wounds has been conducted [48,49,50,51,52]. PAA is considered as a safe substance by the FDA when used in certain food and beverage applications, such as pH regulators and sequestrants in food, as well as an excipient in pharmaceuticals [53]. In addition, it contains carboxylic acid groups, leading to its pH-responsiveness, and protonation of these groups causes muco-adhesion [54]. This last feature makes PAA especially advantageous for skin-mediated controlled drug delivery compared to other polymers [55,56]. Moreover, PAA can undergo the typical reactions of carboxyl to conjugate with other functional groups, obtaining multifunctional derivatives. Furthermore, AA has been used for copolymerization with the NIPAAm monomer to prepare PAA-*co*-PNP copolymers as drug carrier [57,58,59]. Prasanna et al. [57] synthesized PAA-*co*-PNP including graft and random copolymers to be applied as in situ forming hydrogels and evaluated their potential applications for ophthalmic drug delivery. Hoare et al. [58] found that the pure PAA-*co*-PNP nanogels had a minimal impact on the viability of myoblasts, fibroblasts, and macrophages in vitro. Furthermore, smaller PAA-*co*-PNP nanogels induced only mild inflammatory responses after injection into the left leg of the rat. Yang et al. [59] showed that the body weight of mice treated with DOX-loaded PAA-*co*-PNP nanogels was close to the control group, and the DOX-induced cardiotoxicity was significantly inhibited, while a significant weight loss and apparent neutrophil accumulation in the heart slices were observed in the group treated with free DOX. The authors demonstrated that DOX delivered by PAA-*co*-PNP nanogels exhibited stronger anticancer activity but less adverse effects.

Therefore, drug carriers based on PNP and PAA derivatives can respond to external or internal stimulus. Moreover, some of these drug carriers are capable of returning to their initial state after removing stimuli, which is extremely helpful in drug loading and sustained release. In this study, we synthesized a pH/thermo-responsive PAA-*b*-PNP block copolymer via a reversible addition–fragmentation chain transfer (RAFT) polymerization, which could endow the copolymer with a well-defined structure and sharp responsive properties. By increasing the temperature above its phase transition temperature, the PAA-*b*-PNP block copolymer would undergo self-assembly to form nanoparticles with the hydrophobic PNP core and hydrophilic PAA segments as the shell. Then, it was applied to encapsulate a hydrophobic anticancer drug CPT as an environmentally-triggered drug delivery system for the first time. The phase separation temperature or cloud point of the synthesized PAA-*b*-PNP copolymer in a DMSO/buffer solution was investigated as the CPT was dissolved in DMSO. Finally, chitosan (CS) was further imposed onto the CPT-loaded NPs for improving biocompatibility and increasing solidity of particles through the formation of polyelectrolyte complex due to the ionic interaction between the positively-charged CS chains and negatively-charged PAA on the particle surface. CS and its derivatives have been widely used as biomaterials for wound dressing, tissue engineering, and drug delivery carriers, owing to its biocompatibility, biodegradability, antibacterial activity, and wound-healing properties [60,61,62,63,64,65]. Structure, morphology, thermo-responsive properties, and biocompatibility of the prepared NPs were all examined. Moreover, the drug-release behavior, cytotoxicity, and cellular uptake of the CPT-loaded NPs under different biological conditions were studied.

## 2. Materials and Methods

### 2.1. Materials

Acrylic acid (AA) and *N*-isopropylacrylamide (NIPAAm) were supplied from Acros Organics (Geel, Flemish Region, Belgium). Both 2-(Dodecylthiocarbonothioylthio)-2-methylpropionic acid (DMP) as a chain transfer agent (CTA) and 2,2′-azobisisobutyronitrile (AIBN) initiator were obtained from Aldrich (St. Louis, MO, USA). An anticancer drug, camptothecin (CPT), was also obtained from Acros Organics. Dimethyl sulfoxide (DMSO) was obtained from J.T. Baker (Philadelphia, PA, USA). Chitosan (CS) (C&B Co., Taipei, Taiwan) was purified according to the previous procedure [66]. The purified CS had an average molecular weight of 24.5 kDa and a degree of deacetylation (DDA, the fraction of amino group on C-2 position) of 0.94. The mouse fibroblast cells (NIH 3T3), human intestinal epithelial cells (Caco-2), non-small human lung carcinoma cells (H460), and human breast cancer cells (MCF-7) were provided by the Bioresource Collection and Research Center, Hsinchu, Taiwan.

### 2.2. Synthesis of Poly(acrylic acid)-b-poly(N-isopropylacrylamide) (PAA-b-PNP)

**Scheme 1:**
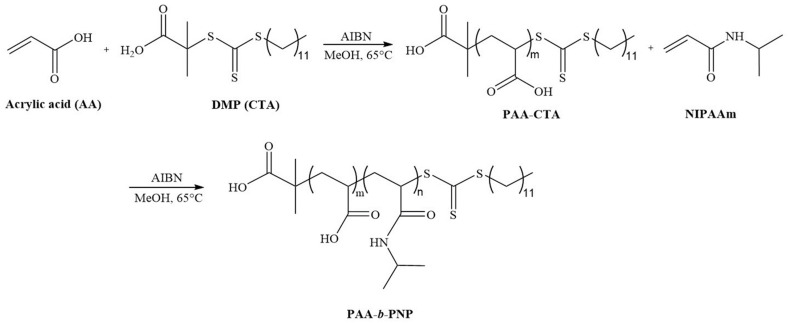
Synthesis of PAA-*b*-PNP block copolymer. The feeding molar ratio of AA/NIPAAm/DMP/AIBN = 21/100/1/0.1.

Reversible addition–fragmentation chain transfer (RAFT) polymerization was applied to synthesize the PAA-*b*-PNP block copolymer. First, AA monomer (0.813 g) was dissolved in 10 mL methanol, and then the solution was added into a reactor. Thereafter, it was purged with nitrogen and heated to 65 °C. DMP (0.196 g) as the chain transfer agent and AIBN initiator (8.8 mg) were dissolved in 10 mL methanol, and the solution was added into the reactor to initiate RAFT polymerization in order to obtain the PAA-CTA macro-chain transfer agent. The molar ratio of AA/DMP/AIBN was fixed at 21/1/0.1. During the reaction, we found that the conversion was nearly complete at 8 h, in which the reaction was found to follow the pseudo-first order kinetics. After 8 h of reaction, the RAFT polymerization of NIPAAm monomer (6.08 g) was continued to obtain PAA-*b*-PNP block copolymer using the PAA-CTA as the chain transfer agent under the same reaction condition, with the exception that the reaction time was shortened to 2 h. The molar ratio of NIPAAm/AA/DMP was fixed at 100/21/1. After the reaction, the solution was cooled down by immersion in an ice-water bath and excess of ice ether was added. The precipitate was separated by centrifugation at 9000 rpm and 4 °C for 15 min and re-dissolved in methanol. The procedure was repeated two times, and the final product was dried at 80 °C to obtain a yellowish PAA-*b*-PNP block copolymer. The reaction scheme is shown in Scheme 1. PAA and PNP homopolymers were also synthesized by RAFT polymerization for comparison.

### 2.3. Preparation of PAA-b-PNP/CPT/CS Nanoparticles by Thermally-Induced Aggregation

The PAA-*b*-PNP copolymer was dissolved in an acetate buffer solution of pH 5.0 (0.1% CH_3_COOH_(aq)_) at a concentration of 1.0 mg/mL. On the other hand, various amounts of CPT (0.30, 0.40, 0.60, and 0.80 mg) were dissolved in 3 mL DMSO. The PAA-*b*-PNP copolymer solution of 8.60 mL was then mixed with the CPT/DMSO solution at room temperature. It was stirred at 100 rpm and heated to 45 °C for inducing self-assembly of the PAA-*b*-PNP to encapsulate the hydrophobic CPT drug. After 30 min of stirring, 1.40 mL CS/acetate buffer solution (0.50 mg/mL, pH 5.0) was added into the dispersion and stirred for another 30 min to obtain PAA-*b*-PNP/CPT/CS nanoparticles. It was centrifuged at 37 °C for 20 min with a speed of 14,000 rpm. The optical absorbance of the supernatant at 369 nm was measured by a microplate reader (Synergy^TM^ H1, BioTek, Winooski, VT, USA) to determine the CPT concentration for calculating the drug-encapsulation efficiency (EE, %) and drug-loaded content (LC, %) from Equations (1) and (2), respectively:

(1) $$Encapsulation\;efficiency\left(EE,\%\right)=\frac{W_{total\;drug}-C_{CPT}\times V_{solution}}{W_{total\;drug}}\times 100$$

(2) $$Loading\;content\left(LC,\%\right)=\frac{EE\%\times W_{total\;drug}}{EE\%\times W_{total\;drug}+W_{carrier}}\times 100$$

The drug-loaded PAA-*b*-PNP/CPT/CS NPs were designated as P_x_CPT_y_C_z_, where P, CPT, and C represented the PAA-*b*-PNP copolymer, CPT drug, and CS at x, y, and z mg, respectively. For comparison, the PAA-*b*-PNP/CPT without CS addition (P_x_CPT_y_) and PAA-*b*-PNP/CS NPs without CPT loading (P_x_C_z_) were also prepared under the same procedure.

### 2.4. Structure Analysis and Physical Properties of Block Copolymer and Nanoparticles

Structure of the synthesized PAA-*b*-PNP copolymer was analyzed by Fourier transform infrared spectrometry (FTIR, iS10, ThermoFisher, Wilmington, MA, USA), nuclear magnetic resonance (NMR, DMX-600, Bruker, Karlsruhe, Germany), and gel permeation chromatography using an isocratic pump PU-4580i (Jasco, Tokyo, Japan) coupled with a refractive index (RI) detector (Shodex, Tochigi Prefecture, Japan) and three serial columns (KF-803, KF-804, and KF-805L, Shodex, Japan). The GPC columns were eluted with chloroform at a flow rate of 0.8 mL/min. For FTIR analysis, transmission mode was applied and it was scanned for 32 times from 4000 to 400 cm^−1^ at a resolution of 4 cm^−1^. For NMR analysis, PAA-CTA and PAA-*b*-PNP were dissolved in D_2_O and d_6_-DMSO, respectively, and placed into an NMR tube to obtain NMR spectrum. For GPC analysis, an eluent of DMF containing 0.1 M LiBr was applied as mobile phase at a flow rate of 0.8 mL/min and the temperature of columns and RI detector was set at 30 °C. The average molecular weights (M_n_, M_w_) and dispersity (Đ) of the polymers were determined based on poly(methyl methacrylate) (PMMA) standard.

Phase transition temperature was determined by observing its cloud point of the synthesized PAA-*b*-PNP copolymer in various solutions. Specifically, the PAA-*b*-PNP copolymer at 8.6 mg was dissolved in several aqueous solutions of 13 mL including two water solutions of pH 5.0 and 7.0 obtained by the addition of 0.10 N HCl_(aq)_ or 0.10 NaOH_(aq)_ in de-ionized water, an acetate buffer solution (pH 5.0), and a mixed solution of an acetate buffer (pH 5.0) with DMSO (10/3, *v*/*v*). Optical transmittance at 450 nm was then measured using a UV-visible spectrophotometer (Helios α, ThermoFisher, USA). The temperature at which the optical transmittance of the PAA-*b*-PNP solution started to decrease was considered as the cloud point.

To understand the interaction between the CPT and PAA-*b*-PNP and the aggregation behavior of the PAA-*b*-PNP, various concentrations of the PAA-*b*-PNP in an acetate buffer solution (pH 5.0) from 1.25 × 10^−4^ to 5.0 × 10^−2^ mg/mL were first prepared. The PAA-*b*-PNP/acetate buffer solution at 5.0 mL was then added with 0.1 mL of CPT/DMSO solution (0.02 mg/mL). After optical excitation at 369 nm, the fluorescence emission spectra of CPT in various solutions were then obtained under 45 °C by using a microplate reader (Synergy^TM^ H1, BioTek, USA). Subsequently, the maximum emission intensity of CPT at 432 nm was plotted versus copolymer concentration. The critical aggregation concentration (CAC) was determined at the point where the rapid decline in emission intensity occurred.

Particle size and polydispersity index (PDI) of the PAA-*b*-PNP, PAA-*b*-PNP/CPT, PAA-*b*-PNP/CS, and PAA-*b*-PNP/CPT/CS were all determined by dynamic light scattering (DLS, Zetasizer Nano, Malvern, UK). Moreover, zeta potential of these NPs was measured by the same instrument. Stability of the PAA-*b*-PNP/CPT/CS NPs at 37 °C was evaluated by measuring the variation of particle size and polydispersity index with time up to 1 month. Morphology of the dispersed particles was observed by using a field emission high-resolution transmission electron microscope (TEM, JEM-2100F-HR, JEOL, Tokyo, Japan).

### 2.5. Drug-Release Behavior

The CPT-loaded NPs were first placed into a dialysis bag (MW_cut-off_ = 12–14 kDa) and suspended in various releasing solutions of HCl_(aq)_ at pH 2.0, and PBS buffer solutions of pH 6.0 and 7.4. Drug release at 37 °C was determined at several specific time intervals, in which a specific amount of sample solution was drawn out for measuring its optical transmittance at 369 nm. The releasing solution was immediately replenished each time at the same amount of sampling solution. The concentration of the released CPT was then determined from the standard calibration curve at the same wavelength. The cumulative release percentage of CPT was calculated using the following equation:

(3) $$Cumulative\;release\left(\%\right)=\frac{C_{i}\times V_{TS}+V_{SS}\times \sum_{j=1}^{i-1}C_{j}}{W_{total\;drug}(mg)}\times 100$$

where $C_{i}$ is the measured CPT concentration (mg/mL) at a specific time interval during drug release, $V_{SS}$ is the sampling volume (mL), $V_{TS}$ is the total solution volume (mL), and $W_{total\;drug}$ is the total weight of the encapsulated CPT drug (mg).

### 2.6. Cellular Compatibility

Cell viability of the empty and CPT-loaded NPs toward normal fibroblast cells was measured by using 3-(4,5-dimethyl-thiazol-2-yl)-2,5-diphenyltetrazolium bromide (MTT) assay. NIH 3T3 cells (1 × 10^4^ cells/well) were seeded onto each well of a 96-well plate. The cells were cultured in an incubator for 24 h at 37 °C. Subsequently, samples were added into each well, and incubated for the next 24 h. The cells without treatment served as the control group. Then, the supernatant was removed, and the cells were washed two times with PBS followed by the addition of 0.10 mL of MTT-containing medium (1 mg/mL MTT). After 4 h of reaction, the formed purple-color product was then dissolved by adding DMSO (1 mL). The absorbance was measured at 570 and 630 nm with a microplate spectrophotometer. The cell viability was determined by the following equation:

(4) $$Cell\;viability(\%)=\frac{\left({OD}_{570,Sample}-{OD}_{630,Sample}\right)}{\left({OD}_{570,Control}-{OD}_{630,Control}\right)}\times 100\%$$

### 2.7. In Vitro Cytotoxicity and Cellular Uptake of the CPT-Loaded Nanoparticles

Caco-2 cancer cells were first cultured in minimum essential medium (MEM, Gibco, Grand Island, CA, USA.) containing 10% fetal bovine serum (FBS), 1% antibiotic-antimycotic solution, 1% non-essential amino acid solution (NEAA), and 1% L-glutamine in an incubator purged with 5% CO_2_ at 37 °C. HT-29 cells were cultured in McCoy’s 5A medium, whereas H460 and MCF-7 were in DMEM medium both containing 10% FBS and 1% antibiotic-antimycotic solution. All cancer cells were then seeded in a 96-well plate at a density of 1 × 10^4^ (Caco-2 and HT-29) and 2 × 10^4^ (H460 and MCF-7) cells per well. After 24 h of culturing at 37 °C, the medium was removed, and the cells were washed two times with Hank’s balance salt solution (HBSS, Sigma, St. Louis, MO, USA.). Thereafter, they were treated with the CPT-loaded NPs at several specific concentrations in HBSS (pH 6.0 or 7.4) and continued culturing for another 6 h. Subsequently, the medium was removed, washed with PBS, and replenished with fresh medium. The cells were further cultured for 24 h, and MTT assay was used to determine the dose-dependent cytotoxicity.

To investigate cellular uptake of the NPs, CS was labeled with a green fluorescence fluorescein isothiocyanate (FITC), and the fluorescent PAA-*b*-PNP/CPT/CS NPs were prepared according to the previously described procedure. The cells were then incubated with the fluorescent NPs at a concentration of 100 μg/mL in the HBSS (pH 6.0 or 7.4) for 3 and 6 h. After incubation, the cells were washed with PBS, followed by paraformaldehyde fixation and propidium iodide (PI) staining. The cells were visualized by a confocal microscope (Eclipse TiE with C2 plus Confocal, Nikon, Tokyo, Japan). For the quantitative analysis, the cells were washed with PBS and subjected to lysis using Triton X-100. The fluorescence intensity of the FITC-labeled NPs from the lytic cells was determined by a micro-plate reader. The cellular-uptake efficiency (%) was defined as the *I_cells_*/*I_control_* × 100 (%), in which the fresh FITC-labeled NPs were applied as the control.

## 3. Results and Discussion

### 3.1. Structural Analysis of the Synthesized PAA-b-PNP Block Copolymer

**Figure 1:**
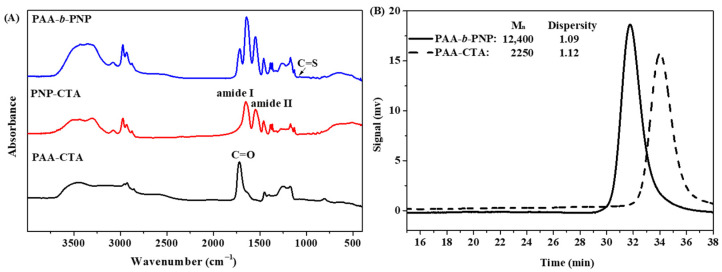
(**A**) FTIR spectra of the PAA-CTA, PNP-CTA, and PAA-*b*-PNP block copolymer, and (**B**) GPC chromatograms of PAA-CTA and PAA-*b*-PNP.

In this study, we synthesized an environmentally-responsive block copolymer of PAA-*b*-PNP as a drug carrier to encapsulate a hydrophobic anticancer CPT. Owing to the addition of hydrophilic AA monomer, the cloud point of PAA-*b*-PNP would depend on the pH value. As a result, this block copolymer was applied not only to encapsulate CPT via thermo-induced self-assembly of PAA-*b*-PNP above its cloud point, but also to deliver the loaded CPT drug triggered by an appropriate pH value. The PAA-*b*-PNP was synthesized by RAFT polymerization, a living radical polymerization in which a block copolymer with a well-defined chain structure could be obtained. First, we synthesized PAA-CTA as a macro-chain transfer agent by reaction of AA monomer and a trithiocarbonate of DMP for 8 h. Subsequently, the PAA-CTA was applied for the RAFT polymerization of PAA-*b*-PNP block copolymer. Structure of the synthesized block copolymer was confirmed by FTIR, NMR, and GPC. Figure 1A shows the FTIR spectra of PAA-CTA, PNP-CTA, and PAA-*b*-PNP copolymer. Notably, the characteristic absorption peaks of PNP block in the copolymer were observed at 1644 cm^−1^ (C=O stretching, amide I), 1549 cm^−1^ (N-H bending, amide II), 1388 and 1368 cm^−1^ (isopropyl group), whereas the main characteristic absorption peak for the PAA block was at 1716 cm^−1^ (C=O stretching in the acid group). Most importantly, a very small absorption peak of the DMP could be seen at 1077 cm^−1^ due to the stretching vibration of C=S. Therefore, FTIR proves the copolymer structure obtained by RAFT polymerization, which is further confirmed by NMR spectra as shown in Supplementary Figures S1 and S2. From the respective peak area ratio of AA and NIPAAm repeating units to the terminal DMP group in the NMR spectra, we were able to calculate the number of repeating units and molecular weights of the PAA-*b*-PNP block copolymer. The synthesized PAA-*b*-PNP copolymer had 21 and 100 repeating units for the PAA and PNP blocks, respectively, which were the same as the corresponding feeding ratio of monomers to the chain transfer agent DMP. Therefore, the corresponding number-average molecular weight (M_n_) of the PAA-*b*-PNP was 13,176 g/mol. The assignments of resonance absorption peaks and detailed calculation can be found in Supplementary Information. The result indicates that under the present reaction conditions, nearly complete conversion of both monomers could be obtained, and the chain structure and composition could be controlled using RAFT polymerization. In addition, Figure 1B shows the GPC traces of the PAA-CTA and PAA-*b*-PNP block copolymer. The calculated molecular weights were close to the values obtained from NMR analysis. Most importantly, the dispersity values (Đ = M_w_/M_n_) of PAA-CTA and PAA-*b*-PNP were found to be 1.12 and 1.09, respectively. Schilli et al. [67] were the first group to synthesize block copolymers of PAA-*b*-PNP by RAFT polymerization, and the obtained copolymer chains had very low dispersity values at 1.03–1.15. Convertine et al. [68] showed that the characteristic linear evolution of M_n_ with conversion and dispersity remained low throughout the RAFT polymerization. Our results also showed a very low dispersity, agreeing to the findings in the literatures and demonstrated the controlled/living nature of the RAFT polymerization.

### 3.2. Cloud Point of the PAA-b-PNP in Various Solutions

**Figure 2:**
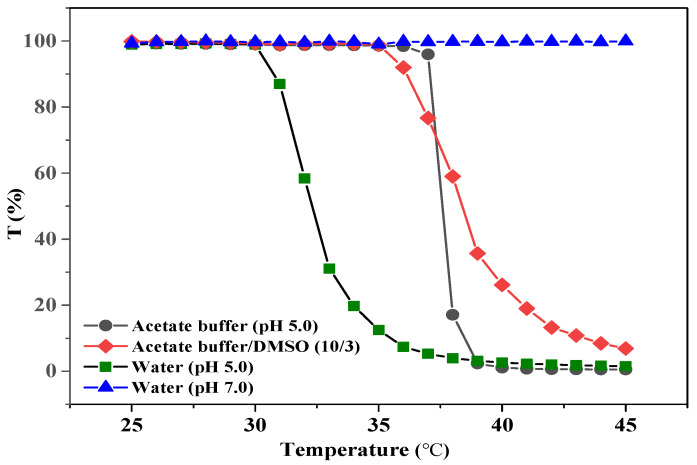
Optical transmittance of the PAA-*b*-PNP block copolymer dissolved in various solutions (8.6 mg/13 mL), including two water solutions of pH 5.0 and 7.0, an acetate buffer solution (pH 5.0), and an acetate buffer/DMSO (10/3, *v*/*v*) solution.

PNP is a well-known thermo-responsive polymer, which has a LCST of 32 °C in water [31]. Copolymerization with acid-containing monomer would endow the resulting copolymer with pH-dependent properties [33]. Consequently, the synthesized PAA-*b*-PNP copolymer would have pH-dependent phase transition temperature, owing to the presence of the acid group. In this study, chitosan was further applied to improve the biocompatibility and rigidity of the prepared NPs. Chitosan is generally dissolved in aqueous acid solutions, whereas anticancer drug CPT can only be dissolved in several organic solvents. Therefore, in this study, we applied an acetate buffer solution and DMSO to dissolve the block copolymer and CPT, respectively; therefore, the variation of phase separation temperature of the synthesized PAA-*b*-PNP in solutions is of interest. Figure 2 shows the optical transmittance of the PAA-*b*-PNP copolymer (8.6 mg) in various solutions (13 mL) of two water solutions of pH 7 and 5, an acetate buffer solution of pH 5, and an acetate buffer added with DMSO (10/3, *v*/*v*). In the water solution of pH 7, we found no precipitation of the PAA-*b*-PNP copolymer up to 50 °C, while the solution remained clear owing to the complete ionization of acid groups in the PAA block. The electrostatic repulsion by the negatively-charged PAA segments prevented the aggregation of copolymer chains. On the other hand, the PAA-*b*-PNP copolymer in the water solution of pH 5 had a cloud point at 31 °C. This is due to the fact that the pH value is close to the pK_a_ of PAA, which was reported between 4.2 and 4.75 [69,70]. Approximately half of the acid groups in the PAA were still protonated, and it is known that the carboxylic acid group is less hydrophilic than its negatively-charged carboxylate group. The pH-dependence of the aggregation degree and phase transition temperature for the PAA-*b*-PNP copolymer have been observed in previous literatures [34,39]. Nevertheless, in the acetate buffer solution of pH 5, the cloud point of the PAA-*b*-PNP copolymer shifted to 37 °C, indicating that the block copolymer became more hydrophilic. It is believed that the acetate buffer might cause a greater ionization of acid groups in the PAA block, and thus increase the cloud point of the copolymer. Moreover, the adsorption of anions on the chain surface would enhance the solubility of PNP chains through the charge−dipole interactions between the adsorbed anions and the water molecules and reduce the extent of PNP aggregation [71,72]. Further addition of DMSO into the acetate buffer shifted the cloud point of the mixture slightly back to 35 °C. Yamauchi and Maeda [73] showed that the PNP/DMSO/water mixtures exhibited LCST phenomenon when the molar fraction of DMSO in the solvent mixture was lower than 0.15. In addition, they found that the LCST decreased with the increase in molar fraction of DMSO from 0.025 to 0.15. They ascribed the decreased LCST to the competition of hydrogen bond with water. The DMSO/H_2_O hydrogen bond through the polar sulfoxide groups with water molecules was stronger than the (PNP)C=O---H_2_O and H_2_O---H-N(PNP) [74]. In addition, the polarization of hydrogen bond by anions could strengthen the interactions between DMSO and PNP (>S=O---H-N(PNP)) [72]. As a result, DMSO removed water molecules from PNP, thereby inducing dehydration of the polymer and reducing the LCST. We believe the same situation occurred in the present PAA-*b*-PNP/DMSO/acetate buffer system. The interplay of environmental conditions, such as the acetate salt, DMSO solvent, and pH value, on the phase transition temperature of the PAA-*b*-PNP block copolymer is complex and deserves further studies.

### 3.3. Fluorescence Study on the Aggregation of Block Copolymer Using CPT as Molecular Probe

**Figure 3:**
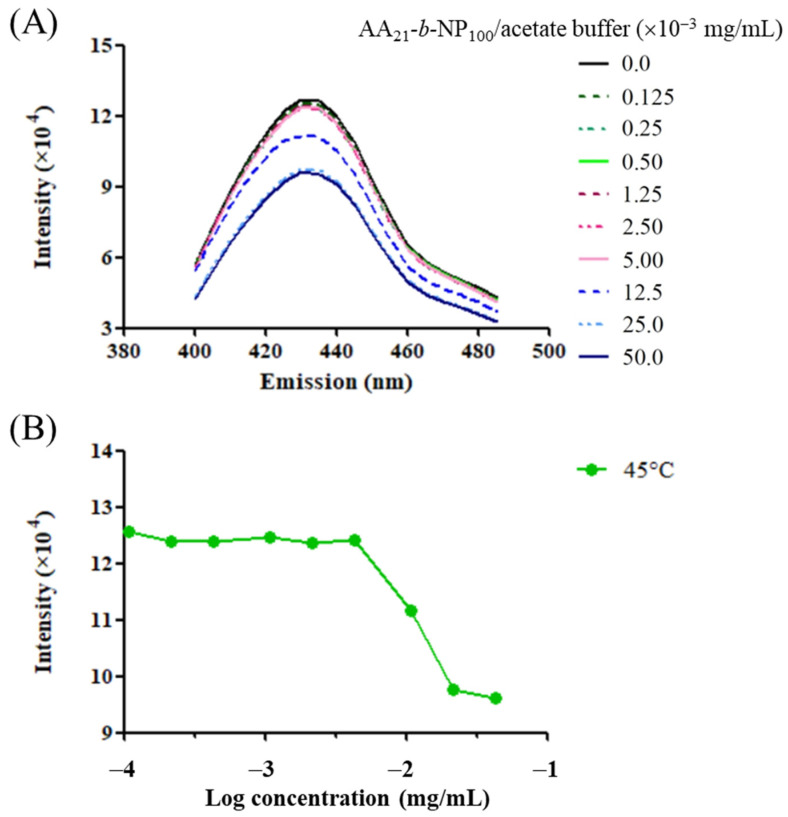
(**A**) Fluorescence spectra of the CPT in various PAA-*b*-PNP/acetate buffer solutions (pH 5.0) at 45 °C, and (**B**) the maximum emission intensity of the CPT at the wavelength of 432 nm in various solutions at different PAA-*b*-PNP concentrations.

CPT is a fluorescent substance with a planar pentacyclic ring structure [75]; therefore, it might be applied as a molecular probe similar to the pyrene, which is sensitive to the polarity of its environment [76,77]. As the concentration of amphiphilic block copolymer is increased to above its critical concentration, the block copolymer starts to aggregate and form a micelle-like structure with a hydrophobic core toward the inside and a hydrophilic shell toward the water. Therefore, the pyrene diffuses into the hydrophobic core, which would exhibit different fluorescence intensities. To understand the self-assembly behavior of the PAA-*b*-PNP and its capability to encapsulate the CPT anticancer drug, various PAA-*b*-PNP/acetate buffer solutions at different concentrations were prepared and added with the CPT/DMSO solution. After optical excitation, the fluorescence spectra of CPT in various solutions were recorded. Figure 3A shows the emission spectra of the CPT in various solutions at 45 °C. This temperature was well-above its cloud point as revealed in the previous section, which ensured that the copolymer could aggregate once the copolymer concentration was above its CAC. The wavelength of maximum emission intensity was found to be about 432 nm for all the spectra, in agreement with the reported value from Chourpa et al. [75]. Then, Figure 3B shows the variation of emission intensity at 432 nm with the PAA-*b*-PNP concentration. At lower copolymer concentrations, the maximum intensity of the CPT maintained a constant value, and then decreased rapidly when the copolymer reached a critical concentration. This indicates that the PAA-*b*-PNP started to form aggregation above this critical concentration (CAC), and the CPT then diffused into the hydrophobic core. The CAC of the block copolymer in the solution was determined as 4.3 μg/mL. This is beneficial for the encapsulation of the CPT in the block copolymer. Moreover, we measured the particle size of the formed NPs in the solutions below and above the critical concentration using DLS. Below the critical concentration, no particles were detected in the solution. On the other hand, an average particle size of 77.4 ± 2.4 nm was measured for the NPs formed in the solution at a block copolymer concentration of 4.5 μg/mL.

### 3.4. Characteristics of the CPT-Loaded PAA-b-PNP/CS Nanoparticles

**Figure 4:**
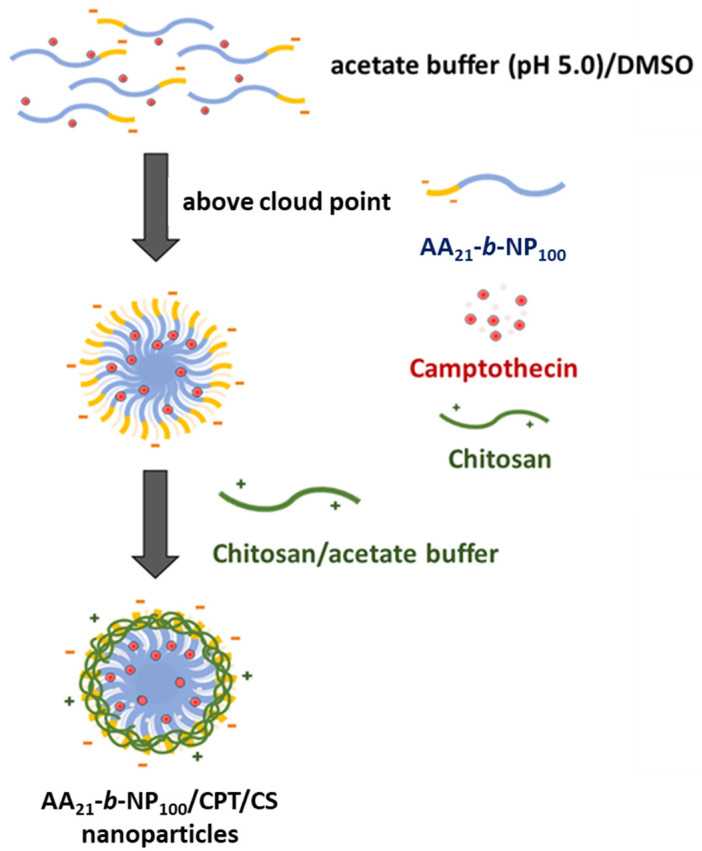
Schematic illustration of the preparation of PAA-*b*-PNP/CPT/CS nanoparticles.

As it was proved in this study, the CPT could be encapsulated by the block copolymer through self-aggregation of the copolymer at temperatures above its cloud point. First, we dissolved the block copolymer in an acetate buffer solution (pH 5.0) at a concentration well-above its CAC, and then it was added with the CPT/DMSO solution. Upon heating to above the cloud point, the block copolymer underwent aggregation, thereby encapsulating the CPT drug in situ. It was expected that the CPT-laden PAA-*b*-PNP particles would have a negative surface charge, owing to the existence of the anionic PAA block on the outer shell. We further added CS/acetate buffer solution (pH 5.0) to endow the particles with a chitosan surface layer through the formation of polyelectrolyte complex between the negatively-charged PAA block (COO^−^) and positively-charged chitosan chains (NH_3_^+^). The formation of polyelectrolyte complex between CS and PAA has been reported in many literatures [78,79,80]. It was proved by FTIR spectra as shown in Supplementary Figure S3. The chitosan (DDA = 0.94) had two prominent peaks at 1638 cm^−1^ (C=O stretching, Amide I) and 1616 cm^−1^ (–NH_2_ bending), in addition to the characteristic absorption peaks of the pyranose ring. The PAA-*b*-PNP copolymer exhibited characteristic absorption peaks of PAA and PNP blocks as already shown in Figure 1. It was observed in Figure S3 that the acid group at 1716 cm^−1^ originally from the PAA block disappeared and another peak at about 1400 cm^−1^ appeared for the PAA-*b*-PNP/CPT/CS NPs, supporting the formation of polyelectrolyte complex from the PAA and chitosan chains. The existence of CS on the surface layer is beneficial for biomedical applications, not only since CS is biocompatible, but also due to the fact that it can transiently open the tight junctions between epithelial cells; therefore, enhancing the paracellular permeability and improving the oral bioavailability of drugs [81,82,83,84]. Figure 4 shows the schematic illustration of the preparation of CPT-loaded NPs. To investigate the effects of the additional amount on the encapsulation efficiency (EE, %) and loading content (LC, %), the added CPT was varied from 0.30 mg to 0.80 mg. Table 1 shows the particle size, polydispersity index (PDI), zeta potential, EE, and LC of various CPT-loaded NPs. It was found that the added CPT at no more than 0.80 mg had no effects on the particle size. They all had similar particle size in the range from 160 to 170 nm. Moreover, they had nearly the same zeta potential at around −31 mV. This is reasonable since all the NPs shown in Table 1 were formed under the same environmental conditions with the same added amounts of block copolymer and chitosan. The negative zeta potential confirmed that the surface layer was mainly composed of the anionic PAA block, while the PNP block resided inside the core. It also demonstrated that the formation of dispersed particles by thermo-induced aggregation was reproducible. Furthermore, both EE and LC increased with the added CPT amount. The EE increased from 8.6% to 29.5% as the CPT was added from 0.3 to 0.8 mg, while the LC increased from 0.28% to 2.53%. We tried to increase the added CPT amount to more than 0.8 mg, yet the CPT became insoluble in the acetate buffer/DMSO solution (10/3, *v*/*v*). The CPT anticancer drug is known to be very hydrophobic. Therefore, the system with the added CPT amount at 0.8 mg (P_8.6_CPT_0.8_C_0.7_) with the highest EE and LC was selected for the following studies.

**Figure 5:**
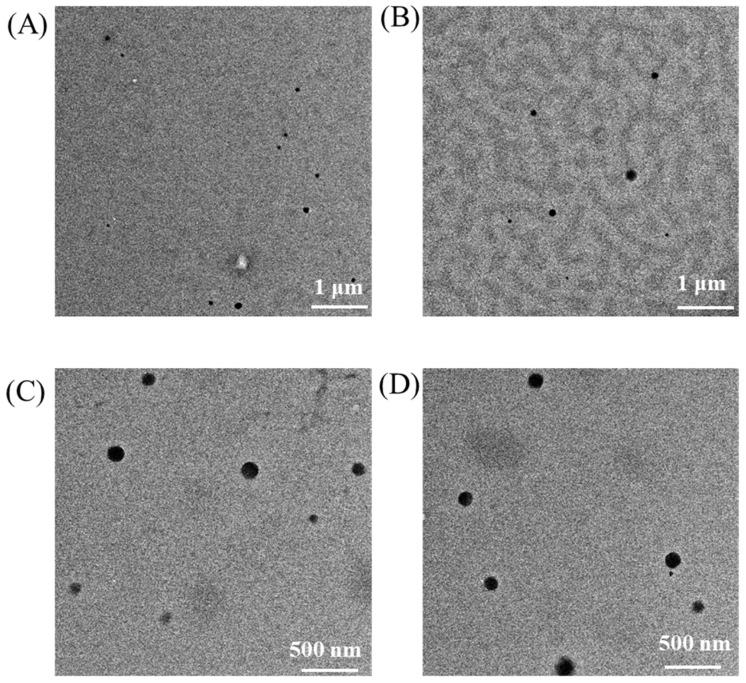
TEM images of the (**A**) PAA-*b*-PNP, (**B**) PAA-*b*-PNP/CPT, (**C**) PAA-*b*-PNP/CS, and (**D**) PAA-*b*-PNP/CPT/CS nanoparticles formed at 37 °C in the acetate buffer (pH 5.0)/DMSO solution (10/3, *v*/*v*). The weight ratio of PAA-*b*-PNP/CPT/CS was 8.6/0.8/0.7.

To understand the development of CPT-loaded NPs in each stage, we tried to measure and compare the particle size and zeta potential of various dispersed particles including PAA-*b*-PNP, PAA-*b*-PNP/CPT, and PAA-*b*-PNP/CPT/CS. Table 2 shows the particle size, polydispersity index, and zeta potential of various particles in the acetate buffer/DMSO solution measured at 37 °C. For the PAA-*b*-PNP NPs formed by increasing the temperature to above its cloud point, an average particle size of about 108 (±9.8) nm was found from DLS (Supplementary Figure S4). The zeta potential was −43.6 mV. The large negative surface potential provided the stability for the NPs suspended in the solution [85]. After CPT encapsulation, the particle size increased to 127.8 (±8.4) nm, whereas the zeta potential decreased to −36.5 mV. Further addition of CS solution into the colloid solution of PAA-*b*-PNP/CPT increased the particle size to about 160.9 (±1.1) nm, owing to the formation of the polyelectrolyte complex between the PAA block and CS on the surface. The addition of positively-charged CS decreased the surface zeta potential to −31.2 mV, as expected. For comparison, we also added the CS solution into the PAA-*b*-PNP dispersion without CPT. Table 2 shows that the average particle size of the formed PAA-*b*-PNP/CS NPs (P_8.6_C_0.7_) was slightly smaller than the dispersed NPs containing CPT (P_8.6_CPT_0.8_C_0.7_) with a decrement of 10 nm. However, the surface zeta potential of P_8.6_C_0.7_ was nearly the same to the value of P_8.6_CPT_0.8_C_0.7_, suggesting the same polyelectrolyte complex formation on the surface. In addition, we only added 0.7 mg of CS as compared to the amount of PAA-*b*-PNP copolymer (8.6 mg) in order to maintain the stability of NPs in the solution as there was an excess amount of negatively-charged COO^−^ group relative to the NH_3_^+^ group. If we increased the added amount of CS, the surface potential would gradually decrease and become positive above a certain amount. For example, when we added the CS at the same amount of block copolymer (PAA-*b*-PNP/CS = 1/1, *w*/*w*), the surface zeta potential became 26.2 mV, while the particle size was greatly increased to 213.9 nm. Moreover, it is interesting to learn that a significantly narrower particle size distribution was found for the NPs with CS than those without CS addition (PDI: 0.046~0.083 vs. 0.20~0.21). The addition of CS not only consolidated the NPs, but also improved the uniformity, owing to the formation of polyelectrolyte complex. It has been proved that smaller particles generally have higher surface charge density [86,87]. Therefore, smaller PAA-*b*-PNP copolymer NPs existing in the system could attract more positively-charged CS chains on their surface, while those larger copolymer NPs attracted less CS chains. As a result, the CPT-loaded NPs became more uniform after the addition of CS. Figure 5 shows the TEM pictures of various NPs formed under different conditions for comparison. It can be seen that they all had a spherical particle shape. Though the average particle size estimated from TEM pictures was smaller than the respective value obtained from DLS measurement as shown in Table 2, their trends of variation from both methods were the same. This is mainly due to the fact that the DLS measured the hydrodynamic diameter of the dispersed particles in solution, while the TEM measured their dried state. Moreover, the core–shell structure could be seen on some particles, supporting the formation of PN core and PAA shell.

### 3.5. Stability and Particle Size of the PAA-b-PNP/CPT/CS Nanoparticles in Various Solutions

**Figure 6:**
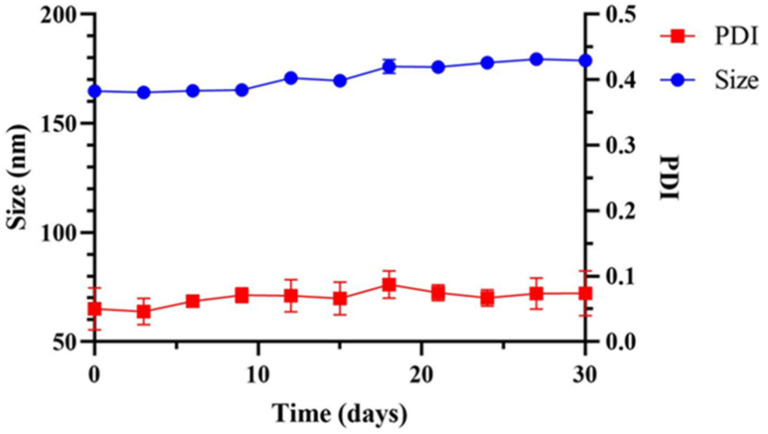
Storage stability of the PAA-*b*-PNP/CPT/CS nanoparticles (P_8.6_CPT_0.8_C_0.7_) in acetate buffer/DMSO solution (10 mL/3 mL) at 37 °C in 30 days.

The poor solubility of CPT in water and in most organic solvents limits its medical application. Therefore, we encapsulated the CPT in the amphiphilic, thermo-responsive copolymer to form NPs dispersed in the aqueous solution. After the formation of PAA-*b*-PNP/CPT/CS particles in the acetate buffer/DMSO solution, we tested their stability at 37 °C up to 1 month. Figure 6 shows that after 1 month of storage, the particle size slightly increases from 164.8 to 178.8 nm, corresponding to an increment of about 8.5%. The dispersion was still stable without any observation of precipitation. The polydispersity index remained below 0.10. Therefore, the almost constant values of particle size and polydispersity index suggest that there was no apparent agglomeration, and the dispersion could be stable at least up to 1 month. This is mainly due to the large surface potential that could prevent the dispersed particles from agglomeration. Hu et al. [72,78] prepared stimuli-responsive chitosan/PAA hollow nanospheres as drug carriers. They found that the electrostatic attraction between the chitosan and PAA could enhance the shell structure of the hollow nanospheres. Moreover, Dai et al. [80] found that the hydrocolloid prepared by glutaraldehyde-crosslinked chitosan was fragile and difficult to work with. However, by the addition of PAA, which could form strong ionic-crosslinking with chitosan, mechanical strength of the hydrocolloid was greatly improved. As a result, the stability of the self-assembled NPs was also improved.

**Figure 7:**
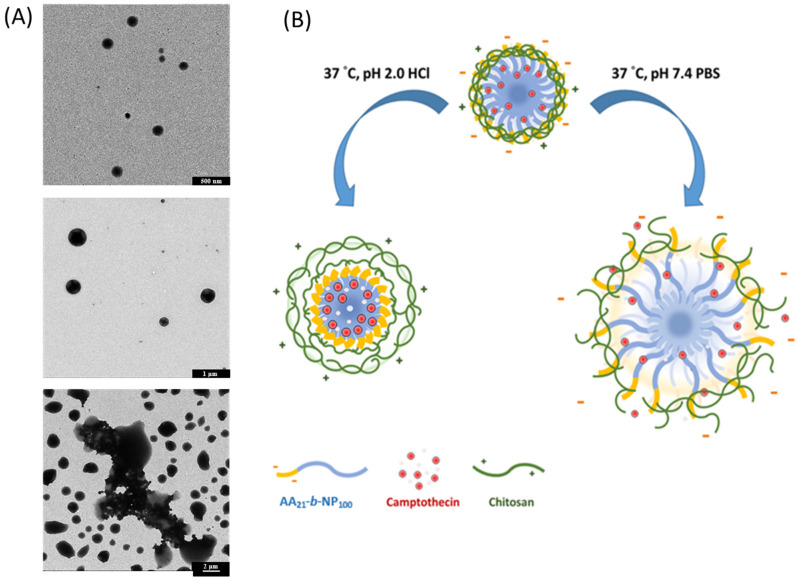
(**A**) TEM images of P_8.6_CPT_0.8_C_0.7_ particles suspended in various solutions of different pH values at 37 °C, top: HCl_(aq)_ (pH 2.0), middle: PBS (pH 6.0), and bottom: PBS (pH 7.4); (**B**) Schematic illustration of the PAA-*b*-PNP/CPT/CS particles in HCl_(aq)_ of pH 2.0 and PBS of pH 7.4.

Though the PAA-*b*-PNP/CPT/CS NPs could be stable in the prepared acetate buffer/DMSO solution, they were also tested under different physiological conditions. It is known that if drugs were administered by the oral route, they would experience a pH gradient as they transit from the stomach (pH 1–2) to the duodenum (pH of about 6), and along the jejunum and ileum (pH 6–7.5) [88]. For simulating gastrointestinal environment, the drug-loaded particles were suspended in several solutions, such as HCl_(aq)_ of pH 2.0 and PBS of pH 6.0 and 7.0. Table 3 shows the particle size, polydispersity index, and zeta potential in these solutions at 37 °C. The particle size was measured as 338.5 nm from DLS when suspended in the HCl_(aq)_ solution of pH 2.0, as two times the size of the original particles in the acetate buffer/DMSO solution. These particles were still very stable, in which the surface zeta potential became positive at 35.1 mV. Clearly, the electrolyte complex of PAA/CS on the surface layer was weakened due to the change in pH value. At pH 2.0, it is believed that nearly all the acid groups in the PAA block were turned into neutral COOH form, and all the amino groups in the CS became positively-charged NH_3_^+^. As a result, the ionic-crosslinking would be greatly decreased, creating a loose surface structure and thereby increasing the particle size. Moreover, the surface potential became positive due to the positively-charged amino groups in CS. On the other hand, the surface potential turned into negative again in the PBS/HCl_(aq)_ buffer solution of pH 6.0. This is due to the fact that increasing the pH value would increase the deprotonation extent of acid groups. At this pH value, the amount of negatively-charged COO^−^ group was apparently greater than the positively-charged NH_3_^+^. In addition, the particle size was increased further to 618.7 nm. Similar result was also found by Nan et al. [89]. They prepared PAA/PNP hydrogel capsules for drug-loading of doxorubicin hydrochloride by using hydroxypropylcellulose/poly(acrylic acid) (HPC/PAA) complexes as the template. As the pH increased from 2.0 to 6.0, the PAA/PNP capsule size increased from 365 nm to 578 nm, which was ascribed to the increased ionization degree of PAA chains and the electrostatic repulsion among the COO^−^ groups of PAA. Finally, in the PBS of pH 7.4, an appreciable increase in the particle size was observed, owing to the further deprotonation of acid groups in the PAA and probably the insolubility of CS. It is known that the pK_a_ of CS is about 6.5 and would not be soluble in aqueous solutions when the pH value is above its pK_a_ [90]. The negatively-charged PAA segments would be very hydrophilic and repel each other due to electrostatic repulsion, and thus extend their chains into the aqueous environment. As a result, the particles were extensively swollen, and some of them were even larger than 1 μm. In fact, this is beneficial for oral administration where the drug-loaded polymeric particles can be stable in the stomach and release the drug in the small intestine by disintegration of particles. Similar result was also found by Fan et al. [91] who prepared NPs based on thiolated chitosan (TCS) and hydroxypropyl methylcellulose phthalate (HPMCP, a polyanion with a pK_a_ of 5.2) by cross-linkages between positively-charged amino groups of TCS and negatively-charged HPMCP. Moreover, they found that increasing the pH value could result in an increase in the particle size of the obtained dispersions and an accompanied decrease in their positive surface charge. Furthermore, due to the pH-sensitive feature of HPMCP, both particle size and zeta potential of NPs were more sensitive to the changing pH value. TEM pictures of the prepared PAA-*b*-PNP/CPT/CS NPs suspended in various solutions of different pH values were also obtained as shown in Figure 7A. It can be seen that uniform particles with nearly spherical shape could be observed in the HCl_(aq)_ of pH 2.0 and PBS/HCl_(aq)_ solution of pH 6.0. Nevertheless, distorted and swollen particles were found in the PBS of pH 7.4, suggesting that the particles became unstable. Figure 7B shows the schematic illustration of the particle structures in the HCl_(aq)_ of pH 2.0 and PBS of pH 7.4, corresponding to the environments of respective stomach and small intestine. Under acidic solution of pH 2.0, the pH-sensitive PAA block became neutral which could form hydrogen bonds with the PNP block through its -COOH groups and amide groups in the PNP. Therefore, the encapsulated CPT drug could be protected from the acidic and/or enzymatic degradation, and thus maintain its bioactivity. On the other hand, the ionic interaction between the PAA block with CS is weakened, leading to the swelling of surface layer and increase in particle size. Nevertheless, the CS remains on the surface of dispersed particles as evidenced from the positive surface potential, probably owing to the entanglements of polymer chains and hydrogen bonds between the CS with the block copolymer. However, increasing the pH value to 7.4 results in the extensively swollen particles, owing to the existence of highly hydrophilic and anionic PAA block. Eventually, disintegration of particles occurs, and the encapsulated drug could be released as in the environment of small intestine.

### 3.6. Drug-Release Behavior

**Figure 8:**
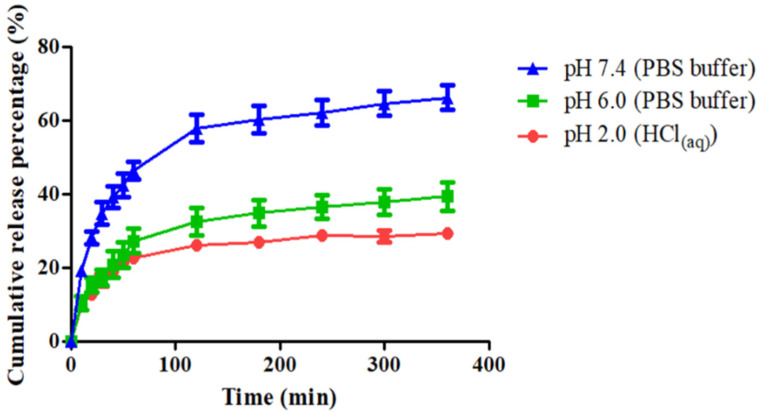
Cumulative release percentage of camptothecin (CPT) from the drug-loaded PAA-*b*-PNP/CPT/CS particles (P_8.6_CPT_0.8_C_0.7_) in various solutions of different pH values at 37 °C. Data were represented by the mean ± SD, n = 3.

Owing to their pH-responsive properties which were examined in previous sections, it was expected that these CPT-loaded particles would also exhibit different drug-release behaviors. Figure 8 shows the drug-release curves of the CPT-loaded particles (P_8.6_CPT_0.8_C_0.7_) suspended in various solutions at 37 °C up to 6 h. It can be seen that increasing the pH value increased the drug-release rate and the ultimate drug-release amount at 6 h. The cumulative release percentage after 6-h release was 29.4% in the HCl_(aq)_ solution of pH 2.0, and it slightly increased to 39.3% in the PBS/HCl_(aq)_ solution of pH 6.0. Under the PBS of pH 7.4, the release rate was greatly increased, and the cumulative release percentage already reached 46.4% after 1-h release. The ultimate release percentage was 66.3% after incubation at 37 °C for 6 h. Therefore, the observed drug-release behaviors were in agreement with the presumed particle structures mentioned previously (Figure 7). In the acidic solution of pH 2.0, the CS chains became positively-charged and more hydrophilic, which could cause the swelling of particles. On the other hand, the PAA block became more hydrophobic and could form hydrogen bonds with the adjacent bonded PNP block, thereby limiting the drug release to a certain extent. In the PBS of pH 7.4, the complete deprotonation of PAA made it highly hydrophilic, and the electrostatic repulsion between the COO^−^ groups of PAA resulted in the extensively swollen particles. As a result, a significant amount of the drug was found to be released at this stage. Xiao et al. [5] developed a series of chitosan-functionalized CPT/curcumin (CUR)-loaded polymeric NPs. The CPT-loading content ranged from 0.6% to 2.8% with the corresponding encapsulation efficiency ranging from 38% to 51%. Approximately 15.5% and 47.6% of the CPT were released from the NPs during the first 4 and 24 h, respectively. Moreover, they demonstrated that the introduction of chitosan to the nanoparticle surface markedly increased cellular-uptake efficiency. Zhang et al. [18] prepared a series of CPT-loaded micelles with the EE and LC ranging from 0.94–6.22% and 10.3–68.6%, respectively. The cumulative release of CPT-loaded micelles at pH 1.2 was 16.5% within 2 h and reached 46% at pH 6.8 within 12 h. Compared to the researches mentioned above, we prepared stable and small P_8.6_CPT_0.8_C_0.7_ NPs with appropriate EE and LC. The release behavior of P_8.6_CPT_0.8_C_0.7_ NPs was in response to the pH value and faster than the other three systems under the same release conditions.

### 3.7. Intracellular Drug Delivery

**Figure 9:**
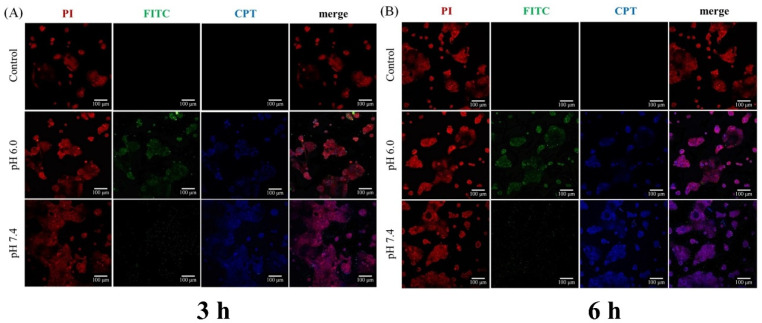
Confocal images of cellular uptake and intracellular distribution of the FITC-labeled nanoparticles and camptothecin (CPT) drug in Caco-2 cells under different pH conditions after incubation for (**A**) 3 h and (**B**) 6 h. Images were presented by propidium iodide (PI) for cells (red), FITC-labeled particles (green), and CPT (blue). The scale bar is 100 μm.

**Figure 10:**
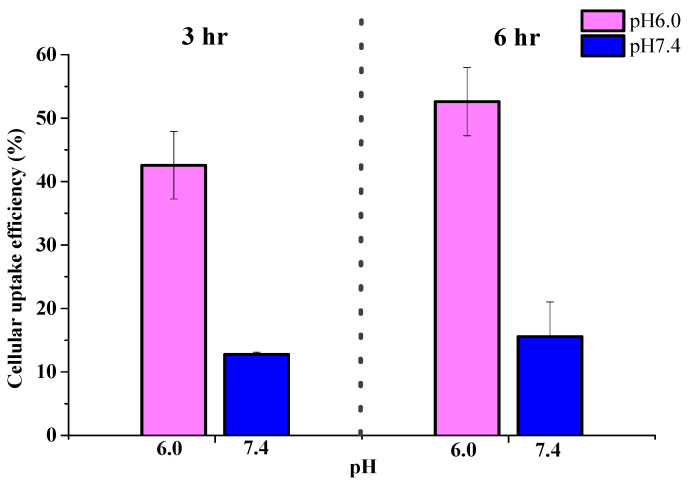
Cellular-uptake efficiency of CPT-loaded nanoparticles in Caco-2 cells incubated at pH 6.0 and 7.4 for 3 and 6 h. Data were represented by the mean ± SD, n = 3.

To examine intracellular distribution of CPT-loaded NPs, CS was labeled with fluorescein isothiocyanate (FITC), which was then applied to prepare fluorescent PAA-*b*-PNP/CPT/CS NPs. As mentioned previously, CPT is a fluorescent compound that can emit light at 432 nm (blue). Figure 9 shows the cellular uptake and intracellular distribution of the FITC-labeled NPs and CPT in Caco-2 cells. Caco-2 is an immortalized cell line of human colorectal adenocarcinoma cells. The cells incubated with the NPs at pH 6.0 for 3 h (Figure 9A) already showed a green fluorescence (NPs) in cytoplasm with their nuclei stained with PI (red fluorescence). Furthermore, a blue fluorescence from CPT was observed within the cells, revealing that the NPs were efficiently internalized by Caco-2 cells and that CPT was delivered into the nuclei. The effective uptake of the NPs by the Caco-2 cells is possibly due to the fact that the NPs are small enough and their surface is covered with CS. Nevertheless, when incubating at pH 7.4, fluorescence of FITC-labeled particles inside the Caco-2 cells was significantly lower than the system co-culturing at pH 6.0 even after 6 h (Figure 9B). Nonetheless, weak green fluorescence around the Caco-2 cells was observed. This is due to the fact that as the pH of the environment increased to 7.4, the CPT-loaded particles became unstable and highly swollen as shown previously in Figure 7. These highly swollen particles could not be internalized by cells, but they could attach to the cells due to the surface chitosan, which has been proven to be a mucoadhesive material and can attach to the Caco-2 cells [83,84,90,91]. Interestingly, strong blue fluorescence from CPT was observed within the cells under culturing at pH 7.4, indicating that the released CPT could penetrate into the cells. A possible explanation is proposed as follows. The CPT-loaded particles could not be taken into the cells at pH 7.4 due to their swollen state and increased particle size. Nevertheless, they could still adhere to the cells due to the presence of chitosan. In addition, as the particles collapsed, the CPT, which was solubilized and stabilized by the block copolymer as evidenced from the previous result (Figure 3), was able to penetrate easily into the Caco-2 cells due to its hydrophobicity. The cellular-uptake efficiencies were further determined by measuring the fluorescence intensity of the FITC-labeled NPs obtained from the inside of the cells and compared to the fluorescence intensity of NPs before culturing. Figure 10 shows that increasing the incubation time from 3 h to 6 h could further increase the cellular-uptake efficiency. In addition, the cellular-uptake efficiency of the CPT-loaded particles was significantly higher when they were incubated at pH 6.0 rather than at pH 7.4, in agreement with the previous observation of Figure 9.

### 3.8. Cytotoxicity

**Figure 11:**
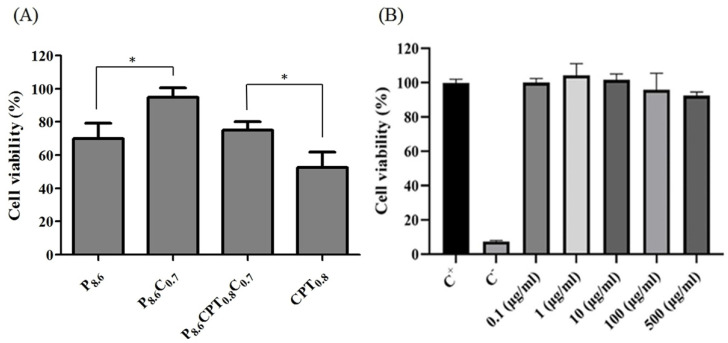
Cell viability of NIH 3T3 cells (**A**) after culturing with various particles of P_8.6_, P_8.6_C_0.7_ P_8.6_CPT_0.8_C_0.7_, CPT, and (**B**) after culturing with nanoparticles (P_8.6_C_0.7_) at different concentrations for 24 h. The control group (C^+^) comprised of the cells without any treatment and the control (C^−^) group comprised of the cells being treated with 10% DMSO solution. Data were compared with the C^+^ group and represented by the mean ± SD, n = 3. * *p* < 0.05.

PNP has been proven to be nontoxic or little toxic to fibroblast cells [45,92,93], while PAA containing acid groups are toxic to the cells [94]. Therefore, it is believed that the block copolymer containing PAA block would exhibit cytotoxicity to a certain extent. Therefore, by surface coating with chitosan to form PAA-*b*-PNP/CS NPs, the biocompatibility should be improved as indicated in Figure 11A (P_8.6_ vs. P_8.6_C_0.7_). Moreover, Figure 11A shows that free CPT was toxic to the 3T3 fibroblast cells. By encapsulation of CPT into PAA-*b*-PNP/CS NPs, the toxicity toward 3T3 cells could be reduced to some extent (CPT vs. P_8.6_CPT_0.8_C_0.7_). Figure 11B shows the cell viability of NIH 3T3 fibroblast cells co-cultured with different concentrations of PAA-*b*-PNP/CS NPs as compared with the control group after 24 h. The result indicates that the NPs did not cause any cytotoxicity toward NIH 3T3. Cell viability percentages for those groups treated with NPs at concentrations up to 500 μg/mL were all above 90%.

**Figure 12:**
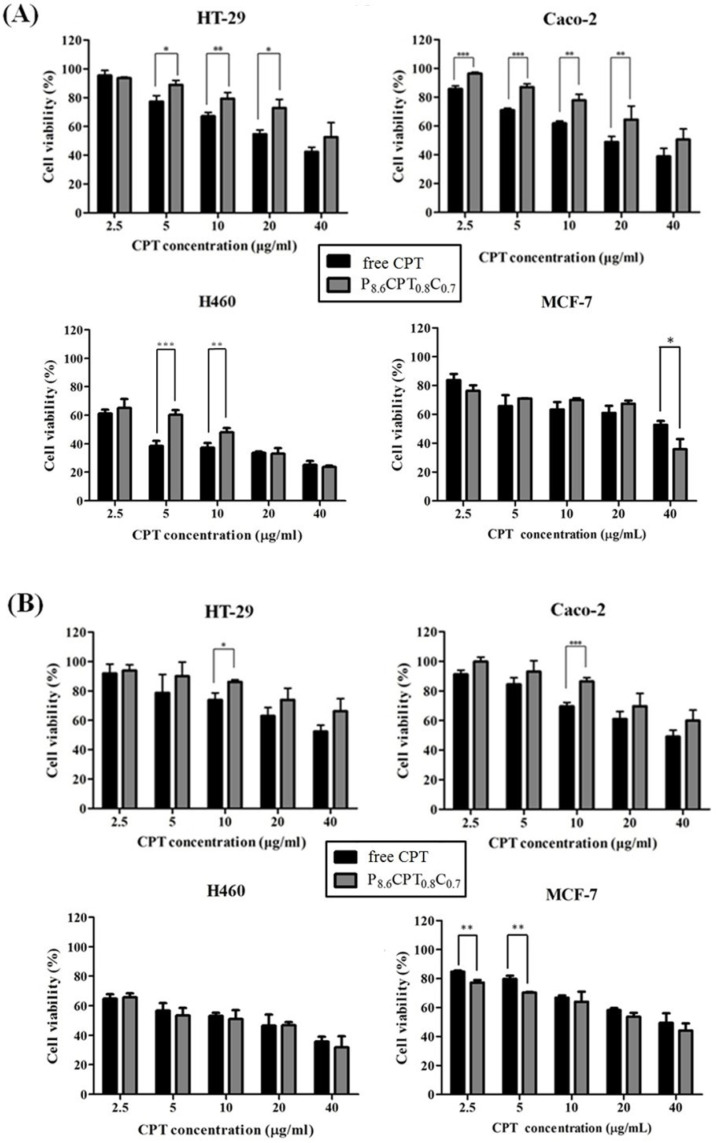
Cell viability of HT-29, Caco-2, H460, and MCF-7 cells being treated with free CPT and P_8.6_CPT_0.8_C_0.7_ for 24 h at the same CPT concentration as compared to the control group without any treatment: (**A**) pH 6.0, (**B**) pH 7.4. Data were represented by the mean ± SD, n = 3. * *p* < 0.05, ** *p* < 0.01, *** *p* < 0.001.

Cytotoxicity of P_8.6_CPT_0.8_C_0.7_ NPs on several cancer cell lines including HT-29, Caco-2, H460, and MCF-7 cells were evaluated in vitro. Figure 12 shows that all cancer cells exhibited dose-dependent cytotoxicity against free CPT and CPT-loaded NPs, and the highest cytotoxicity was observed for H460 cells. In addition, the cytotoxicity was slightly higher when the cells were under incubation at pH 6.0 than at pH 7.4. This is reasonable as the cellular-uptake efficiency of the CPT-loaded NPs was higher at pH 6.0 rather than at pH 7.4 as shown previously in Figure 9 and Figure 10. Moreover, the slightly acidic environment favors the inhibition of cell proliferation by CPT. This might be due to the different equilibrium ratios between the lactone ring (closed form) and open ring in CPT under different pH environments [6,7,10]. The lactone ring, being active as a topoisomerase inhibitor, is favored in acidic condition. This is advantageous as the microenvironment of many cancer cells is found to be acidic. Furthermore, free CPT was slightly more cytotoxic in cells compared to the CPT-loaded NPs under incubation at pH 6.0 as shown in Figure 12A. It is suspected that the cell viability of free-CPT group could be affected by DMSO since the CPT was dissolved in DMSO and diluted to appropriate concentrations in the medium for co-culture. Xiao et al. [5] pointed out that the presence of DMSO in the free-drug solution could assist in the penetration of free drug into cells and DMSO itself exerted a toxic effect that was superimposed on the free drug. Another reason might be that CPT was released gradually from the NPs that were taken up by cells via endocytosis, causing a lower cytotoxicity in the cancer cells than free CPT. Considering that only 39.3% of the loaded CPT was released in the buffer solution of pH 6.0 and 66.3% in the PBS of pH 7.4 after 6 h (Figure 8), the difference in cytotoxicity between free CPT and drug-loaded NPs could be clearly understood. On the other hand, this also proves the successful cellular uptake and drug release from NPs in response to intracellular microenvironment. Furthermore, we found that the cytotoxicity of NPs varied among different cells. For MCF-7 cells, the CPT-loaded NPs exhibited higher cytotoxicity than free CPT at concentrations up to 40 μg/mL. When under culturing at pH 7.4, it can be seen that the difference in cell viability percentages for free CPT and CPT-loaded NPs was not very significant (Figure 12B). It is known that, for clinical chemotherapy, cancer cells in tumors are not likely subjected to a long-term treatment with free CPT due to the lack of passive targeting to tumor sites [95]. In addition to its poor solubility and clearance by reticuloendothelial system, free CPT undergoes water hydrolysis and becomes deactivated rapidly under physiological conditions, forming inactive CPT via the lactone ring-opening process [12]. Moreover, it has a strong interaction with blood plasma proteins [96]. Therefore, in vivo animal experiments for pharmacokinetic analysis should be undertaken for comparison. In conclusion, the prepared PAA-*b*-PNP/CS NPs could encapsulate the highly hydrophobic CPT drug and protect it from the acidic environment in stomach. The CPT-loaded NPs could be internalized by Caco-2 cancer cells in a slightly acidic environment and release the CPT drug for effectively inhibiting the replication of DNA, as in many cancer cell microenvironments.

## 4. Conclusions

In order to encapsulate a hydrophobic drug, such as CPT, an environmentally-responsive block copolymer of PAA-*b*-PNP was applied as the drug carrier in this study. The copolymer was synthesized by RAFT polymerization, which was composed of 21 AA repeating units and 100 NIPAAm repeating units. To prepare drug-loaded NPs, DMSO was applied to dissolve the CPT, and then mixed with the block copolymer in an acetate buffer solution. The cloud point of this PAA-*b*-PNP in the acetate buffer/DMSO (10/3, *v*/*v*) at a concentration of 8.6 mg/13 mL was determined as 35 °C. By increasing the temperature to well-above its cloud point, the block copolymer could undergo self-aggregation to form NPs, and simultaneously encapsulate the CPT drug inside the PNP core due to the hydrophobic interaction between the CPT and PNP block as evidenced by fluorescence spectrometry. To improve the biocompatibility and increasing solidity of particles, CS was adhered to particle surface through the formation of polyelectrolyte complex with the PAA shell. The PAA-*b*-PNP/CS NPs had good biocompatibility toward NIH 3T3 cells. Moreover, the CPT-loaded NPs exhibited different drug-release behaviors and cellular uptakes under different biological conditions. Under an acidic environment of pH 2.0, the PAA-*b*-PNP/CPT/CS NPs maintain their uniform spherical particles with an average particle size of 229 nm as determined from TEM pictures. They could protect the CPT from this acidic environment of pH 2.0. Moreover, in a slightly acidic environment of pH 6.0, these NPs could be internalized by the Caco-2 cancer cells followed by intracellular release of the CPT. However, they were highly swollen at pH 7.4 and attached to the Caco-2 cells due to their surface chitosan. The released CPT from the swollen NPs could then diffuse into the Caco-2 cells. Cancer cells including HT-29, Caco-2, H460, and MCF-7 exhibited dose-dependent cytotoxicity against free CPT and CPT-loaded NPs, and the highest cytotoxicity was observed for H460 cells. Therefore, the dual thermo- and pH-responsive NPs have the potential to be applied as the drug carrier for hydrophobic drugs.

## Figures and Tables

**Table 1:** Effects of the added CPT amount on the particle size, polydispersity index (PDI), zeta potential, encapsulation efficiency (EE, %), and loading content (LC, %) of the PAA-*b*-PNP/CPT/CS nanoparticles formed in the acetate buffer (pH 5.0)/DMSO solution (10/3, *v*/*v*) *.

Nanoparticles	P_8.6_CPT_0.3_C_0.7_	P_8.6_CPT_0.4_C_0.7_	P_8.6_CPT_0.6_C_0.7_	P_8.6_CPT_0.8_C_0.7_
Size (nm)	170.1 ± 2.2 **	162.2 ± 4.7	162.1 ± 2.0	168.4 ± 3.4
PDI	0.049 ± 0.005	0.059 ± 0.012	0.034 ± 0.003	0.042 ± 0.019
Zeta potential (mV)	−31.6 ± 0.4	−30.3 ± 0.7	−31.1 ± 0.9	−30.6 ± 0.7
EE (%)	8.6 ± 2.3	11.0 ± 1.1	21.3 ± 1.4	29.5 ± 0.9
LC (%)	0.28 ± 0.07	0.48 ± 0.05	1.37 ± 0.09	2.53 ± 0.07

* The PAA-*b*-PNP/CPT/CS nanoparticles were denoted as P_x_CPT_y_C_z_, where P, CPT, and C represented the PAA-*b*-PNP copolymer, camptothecin, and chitosan at x, y, and z mg, respectively. ** Data were represented by the mean ± SD, n = 3.

**Table 2:** Particle size, polydispersity index (PDI), and zeta potential of dispersed particles formed in the acetate buffer (pH 5.0)/DMSO solution (10/3, *v*/*v*) and measured at 37 °C.

Nanoparticles *	P_8.6_	P_8.6_CPT_0.8_	P_8.6_C_0.7_	P_8.6_CPT_0.8_C_0.7_
Size-DLS (nm)	108.0 ± 9.8 **	127.8 ± 8.4	150.2 ± 1.7	160.9 ± 1.1
Size-TEM (nm)	71.2 ± 18.3	96.9 ± 42.9	127.7 ± 52.3	130.0 ± 33.4
PDI	0.21 ± 0.01	0.20 ± 0.02	0.046 ± 0.025	0.083 ± 0.016
Zeta potential (mV)	−43.6 ± 2.2	−36.5 ± 2.7	−32.5 ± 1.1	−31.2 ± 0.4

* P_x_, CPT_y_, and C_z_ represented the PAA-*b*-PNP copolymer, camptothecin, and chitosan at x, y, and z mg, respectively. ** Data were represented by the mean ± SD.

**Table 3:** Particle size, polydispersity index (PDI), and zeta potential of the P_8.6_CPT_0.8_C_0.7_ particles in various solutions of different pH values at 37 °C.

Solution	HCl_(aq)_ (pH 2.0)	PBS (pH 6.0)	PBS (pH 7.4)
Size-DLS (nm)	338.5 ± 12.1	618.7 ± 51.7	1164.0 ± 174.2
Size-TEM (nm)	229.4 ± 72.5	556.7 ± 133.4	1237.8 ± 211.8
PDI	0.074 ± 0.018	0.323 ± 0.098	0.132 ± 0.115
Zeta potential (mV)	35.1 ± 2.1	−13.2 ± 0.2	−13.2 ± 0.2

## Data Availability

The raw/processed data required to reproduce these findings cannot be shared at this time due to technical or time limitations.

## Supplementary Materials

The following supporting information can be downloaded at: https://www.mdpi.com/article/10.3390/polym15112463/s1, Figure S1: NMR spectrum of the PAA-CTA using D_2_O solvent. Figure S2: NMR spectrum of the PAA-*b*-PNP block copolymer using *d_6_*-DMSO. Note: the signals of MeOH and DMSO in the NMR spectrum were caused by the little residue from the solvents. Figure S3: FTIR spectra of chitosan, PAA-*b*-PNP copolymer, and P_8.6_CPT_0.8_C_0.7_ nanoparticles. Figure S4: Dynamic light scattering profiles of P_8.6_, P_8.6_CPT_0.8_ and P_8.6_CPT_0.8_C_0.7_ NPs.
