# Supplementary material for: In Situ Encapsulation of Camptothecin by Self-Assembly of Poly(acrylic acid)-b-Poly(N-Isopropylacrylamide) and Chitosan for Controlled Drug Delivery
Source: Polymers (Basel). 2023 May 26;15(11):2463. doi: 10.3390/polym15112463 (PMC10255083; doi:10.3390/polym15112463)
Supplement: Supplementary file 1 [file polymers-15-02463-s001.zip › polymers-2367038-supplementary.pdf]

Supplementary 1

In Situ Encapsulation of Camptothecin by Self-Assembly of Poly(acrylic acid)-*b*-Poly(*N*-Isopropylacrylamide) and Chitosan for Controlled Drug Delivery

Yi-Cheng Huang [1], Yang-Jie Zeng [1], Yu-Wei Lin [2], Hung-Chih Tai [1] and Trong-Ming Don [2,*]

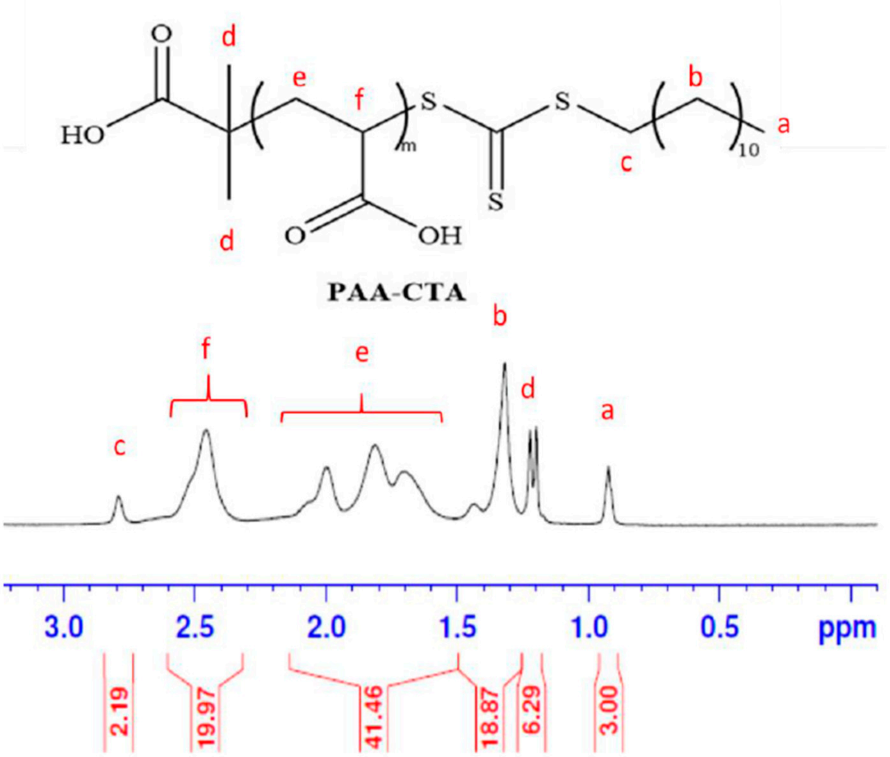

Figure S1. NMR spectrum of the PAA-CTA using D_2O solvent.

NMR spectrum: A small absorption peak at 0.90 ppm was ascribed to the terminal methyl group (-CH_3) of the DMP at the chain end. The doublet peaks from 1.15 to 1.25 ppm were caused by the two CH_3 groups on the other side of the DMP (>C(CH_3)$_2$). The peak from 1.25 to 1.5 ppm was due to the 10 methylene groups in DMP (-S-CH_2-(CH_2)$_{10}$-CH_3). Two broad absorption peaks at 1.5−2.1 ppm and 2.35−2.55 ppm were due to the respective CH_2 and CH groups in the repeating units of PAA-CTA. The small absorption peak from 2.75 to 2.85 ppm was due to the >CH_2 group adjacent to the trithiocarbonate in the DMP (-S-(C=S)-S-CH_2-(CH_2)$_{10}$-CH_3).

To calculate the degree of polymerization (m) of PAA-CTA, two methods were applied as follows:

(1) The total peak area of the methylene (CH_2) and methine (CH) groups in the repeating unit of PAA (3 m hydrogens) was divided by the area of the terminal methyl group in the DMP (3 hydrogens), as shown in the following equation:

$$\overline{X_{n,AA}} = \text{m}; \ \frac{3\text{m}}{3} = \frac{\left(I_{\text{CH}_2} + I_{\text{CH}}\right) \, in \, PAA}{I_{\text{CH}_3} \, in \, DMP}$$

$$m = \frac{41.46 + 19.97}{3.00} = 20.5$$

(2) The total peak area of the methylene (CH_2) and methine (CH) groups in the repeating unit of PAA (3 *m* hydrogens) was divided by the total area of the terminal methyl group, two methyl groups in isopropyl, and ten methylene groups in the DMP (29 hydrogens):

$$\frac{3m}{29} = \frac{(I_{CH_2} + I_{CH})\ in\ PAA}{(I_{CH_3} + I_{(CH_3)_2} + I_{(CH_2)_{10}})\ in\ DMP}$$

$$\frac{3m}{29} = \frac{41.46 + 19.97}{3.00 + 6.29 + 18.87};\ m = 21.1$$

Both calculated values from methods (1) and (2) were close to 21, which were exactly the same as the feeding molar ratio of AA monomer to the DMP chain transfer agent, proving that the conversion could reach nearly 100% after 8 h of reaction.

Therefore, the number-average molecular weight of PAA-CTA was as follows:

$$\overline{M_n} = m \times MAA + MDMP = 21 \times 72 + 364 = 1,876$$

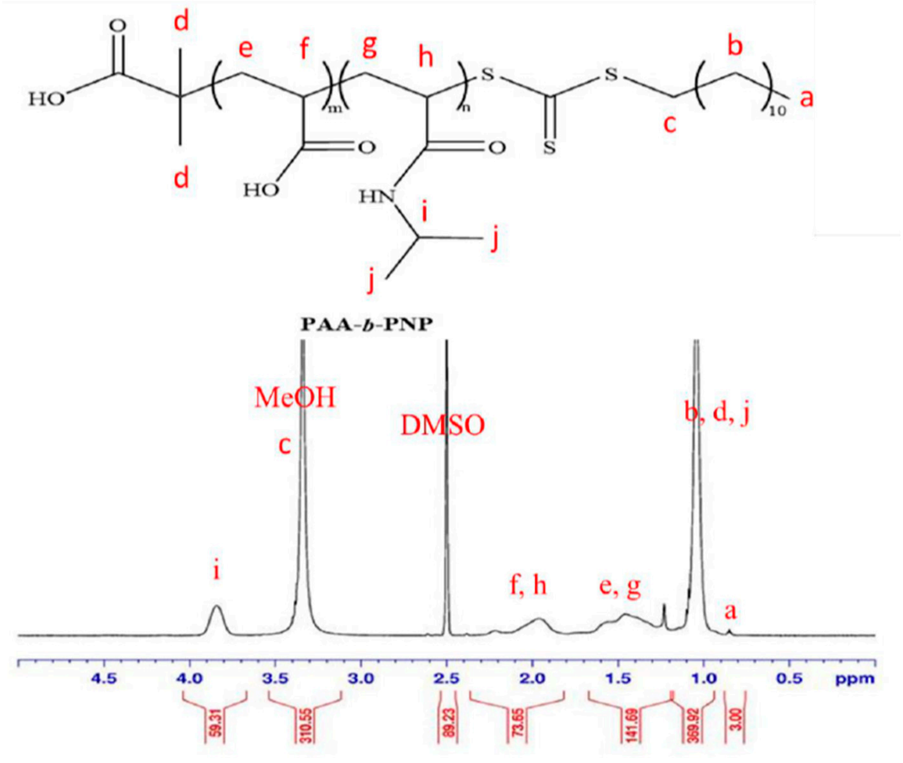

Figure S2. NMR spectrum of the PAA-*b*-PNP block copolymer using *d*6-DMSO. Note: The signals of MeOH and DMSO in the NMR spectrum were caused by the little residue from the solvents.

NMR spectrum: A small absorption peak at 0.85 ppm was ascribed to the terminal methyl group ($-CH_3$) of the DMP. The adjacent absorption peak from 0.90 to 1.20 ppm was due to the two CH_3 groups in the isopropyl of PNP block ($-CH(CH_3)_2$) with a minor contribution from the DMP ($>C(CH_3)_2$, $-S-CH_2-(CH_2)_{10}-CH_3$). Two broad absorption peaks at 1.25−1.75 and 1.80−2.30 ppm were caused by the respective CH_2 and CH groups in the repeating units of PAA-*b*-PNP copolymer. The absorption peak from 3.2 to 3.45 ppm was due to the residual methanol

solvent used in the reaction and the CH_2 adjacent to the trithiocarbonate in the DMP (-S(C=S)-S-CH_2-$(CH_2)_{10}$-CH_3). The absorption peak at 3.85 ppm was due to the CH absorption in the isopropyl group of PNP block (N-CH-$(CH_3)_2$).

To calculate the degree of polymerization, we applied the method similar to the previous method (2). The terminal methyl peak in DMP as applied in method (1) was quite small, which would cause a large amount of uncertainty in the calculation. First, we calculated the degree of polymerization *n* in the PNP block using the following equation, in which the total peak area (6*n* + 29 hydrogens), composed of the two methyl groups in the isopropyl of PNP block and the terminal methyl group, two methyl groups in isopropyl, and ten methylene groups in the DMP, was divided by the peak area of the CH absorption in the isopropyl group of PNP block (*n*), as follows:

$$\overline{X_{n,NP}} = \mathrm{n};\ \frac{6\mathrm{n}+29}{\mathrm{n}} = \frac{I_{(\mathrm{CH_3})_2}\ in\ PNP + (I_{\mathrm{CH_3}} + I_{(\mathrm{CH_3})_2} + I_{(\mathrm{CH_2})_{10}}\ in\ DMP}{I_{\mathrm{CH}}\ in\ isopropyl\ of\ PNP}$$

$$\frac{6\mathrm{n}+29}{\mathrm{n}} = \frac{369.92+3}{59.31};\ \mathrm{n} = 100.8$$

The degree of polymerization of PNP was found to be close to the feeding molar ratio of NIPAAm monomer to the DMP chain transfer agent. Subsequently, we calculated the degree of polymerization *m* in the PAA block using the following equation. The total peak area of the methylene and methine in both of the repeating units of PAA and PNP blocks (3*n* + 3*m*) was divided by the area of the CH absorption in the isopropyl group of PNP block (*n*), in which *n* was 100.8 as obtained previously:

$$\frac{3\mathrm{n}+3\mathrm{m}}{\mathrm{n}} = \frac{\left(I_{\mathrm{CH_2}} + I_{\mathrm{CH}}\right)\ in\ both\ PAA\ and\ PNP}{\mathrm{I_{CH}\ in\ isopropyl\ of\ DMP}}$$

$$\frac{3\mathrm{n}+3\mathrm{m}}{\mathrm{n}} = \frac{141.69+73.65}{59.31};\ \mathrm{n} = 100.8;\ \mathrm{m} = 21.2$$

Therefore, the number-average molecular weight of PAA-*b*-PNP is as follows:

$$\overline{\mathrm{M_n}} = \mathrm{m} \times M_{AA} + M_{NIPAAm} + M_{DMP} = 21 \times 72 + 100 \times 113 + 364 = 13{,}176$$

A value of 21.2 was obtained for the *m* when substituting *n*= 100.8 into the equation. This value was almost the same as the value of the previous PAA-CTA. This indicates that the activity of the macro-chain transfer agent PAA-CTA could be maintained and the following polymerization of PNP block could be proceeded with a complete conversion under the present reaction condition. In conclusion, we sucessfully synthesized a block copolymer of PAA-*b*-PNP with a controlled structure and composition simply by controlling the feeding molar ratio of monomers to the chain transfer agent using RAFT polymerization.

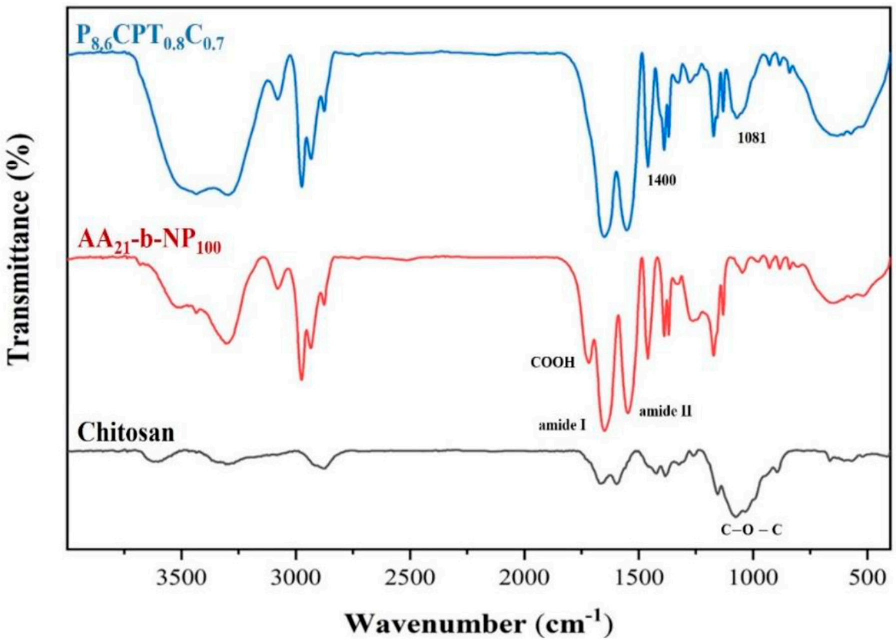

Figure S3. FTIR spectra of chitosan, PAA-*b*-PNP copolymer, and $P_{8.6}CPT_{0.8}C_{0.7}$ nanoparticles. $P_{8.6}CPT_{0.8}C_{0.7}$ nanoparticles were prepared by the addition of 3 mL CPT/DMSO (0.1 mg/mL) solution to 8.6 mL of PAA-*b*-PNP/acetic acid buffer solution (1.0 mg/mL, pH 5), heating to 45 °C to induce nanoparticle formation, and finally adding 1.4 mL CS/acetate buffer solution (0.5 mg/mL) to the colloid solution. P, CPT, and C represented PAA-*b*-PNP copolymer, CPT drug, and chitosan, respectively.

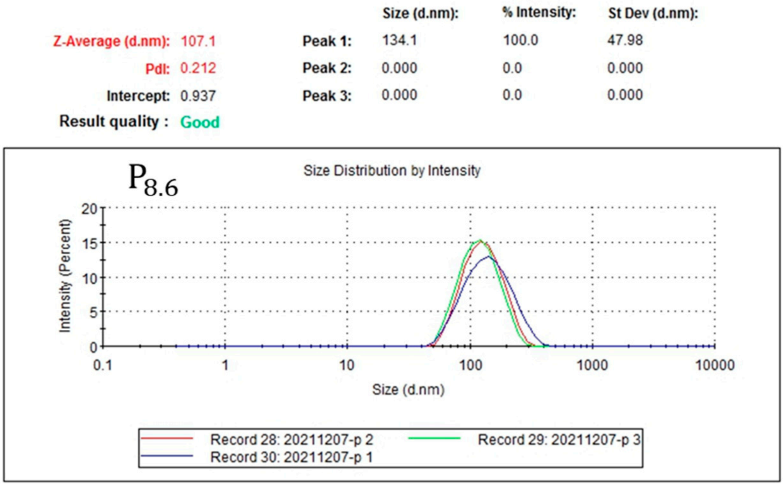

			Size (d.nm):	% Intensity:	St Dev (d.nm):
Z-Average (d.nm):	129.1	Peak 1:	160.9	100.0	57.73
PdI:	0.211	Peak 2:	0.000	0.0	0.000
Intercept:	0.949	Peak 3:	0.000	0.0	0.000
Result quality :	Good				

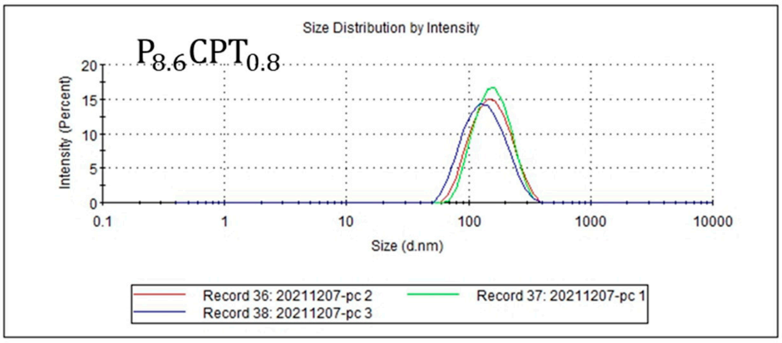

			Size (d.nm):	% Intensity:	St Dev (d.nm):
Z-Average (d.nm):	156.8	Peak 1:	168.8	100.0	47.10
PdI:	0.065	Peak 2:	0.000	0.0	0.000
Intercept:	0.946	Peak 3:	0.000	0.0	0.000
Result quality :	Good				

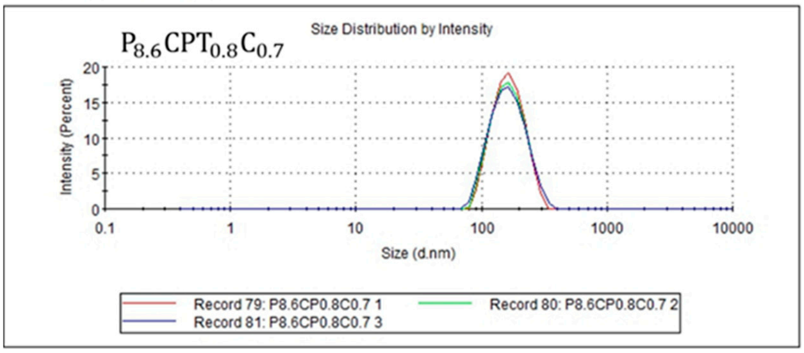

Figure S4. Dynamic light scattering profiles of $P_{8.6}$, $P_{8.6}CPT_{0.8}$, and $P_{8.6}CPT_{0.8}C_{0.7}$ NPs.
